# Feasibility of community-based HIV self-screening in South Africa: a demonstration project

**DOI:** 10.1186/s12889-019-7122-5

**Published:** 2019-07-08

**Authors:** Limakatso Lebina, Ntombexolo Seatlholo, Noah Taruberekera, Mopo Radebe, Anthony Kinghorn, Tessa Meyer, Miriam Mhazo, Kennedy Otwombe, Khuthadzo Hlongwane, Ashley Ringane, Nthabiseng Koloane, Mbali Nkuta, Nkhensani Nkhwashu, Thato Farirai, Patience Kweza, Thato Chidarikire, Simukai Shamu, Tendesayi Kufa, Adrian Puren, Neil Martinson, Minja Milovanovic

**Affiliations:** 1 Perinatal HIV Research Unit (PHRU), SA MRC Soweto Matlosana Collaborating Centre for HIV/AIDS and TB, Faculty of Health Sciences, University of the Witwatersrand, Johannesburg, South Africa; 2 Society for Family Health, Johannesburg, South Africa; 3 Foundation for Professional Development, Tshwane, South Africa; 4 HIV and AIDS and STI Cluster, National Department of Health, Pretoria, South Africa; 5 Centre for HIV and STIs, National Institute for Communicable Diseases, Johannesburg, South Africa; 6 School of Public Health, University of Witwatersrand, Johannesburg, South Africa; 7 Division of Virology, School of Pathology, University of the Witwatersrand, Johannesburg, South Africa; 8 Center for TB Research, Johns Hopkins University, Baltimore, MD USA

**Keywords:** HIV self-test, High risk populations, HIV, Demonstration project

## Abstract

**Background:**

HIV diagnosis is a critical step in linking HIV-infected individuals to care and treatment and linking HIV-uninfected persons to prevention services. However, the uptake of HIV testing remains low in many countries. HIV self-screening (HIVSS) is acceptable to adults, but there is limited data on HIVSS feasibility in community programmes. This study aimed to evaluate the feasibility of HIVSS in South Africa.

**Methods:**

We conducted a prospective study that enrolled participants through mobile site, homebased, workplace and sex worker programmes in two townships from May to November 2017. Following an information session on HIVSS, interested participants were offered one of three methods of HIVSS testing: supervised, semi-supervised, and unsupervised. Participants who opted for unsupervised testing and those who tested HIV positive after semi- or supervised HIVSS were followed up telephonically or with a home visit one week after receipt of the test kit to confirm results and linkages to care. Follow-up visits were concluded when the participant indicated that they had used the kit or had accessed a confirmatory HIV test.

**Results:**

Of the 2061 people approached, 88.2% (1818/2061) received HIV testing information. Of this group, 89% (1618/1818) were enrolled in the study and 70.0% (1133/1618) were tested for HIV with the kit. The median age was 28 (IQR:23–33) years with an even gender distribution. Of those enrolled, 43.0% (696/1618) were identified through homebased outreach, 42.5% (687/1618) through mobile sites, 7.3% (118/1618) at their workplace and 7.2% (117/1618) from sex worker programmes. A total of 68.7% (1110/1616) selected unsupervised HIVSS, whereas 6.3% (101/1616) opted for semi-supervised and 25.0% ((405/1616) chose supervised HIVSS. Overall, the HIV prevalence using the HIVSS test was 8.2% (93/1129). Of those newly diagnosed with HIV, 16% (12/75) were initiated on ART. Almost half (48.0%; 543/1131) of those tested were linked to a primary HIV test as follows: supervised (85.2%; 336/394); semi-supervised (93.8%; 91/97) and unsupervised (18.1%; 116/640).

**Conclusion:**

Unsupervised HIVSS was by far the most selected and utilised HIVSS method. Linkages to primary and confirmatory testing for the unsupervised HIVSS and further care were low, despite home visits and telephonic reminders.

## Background

Timely detection of human immunodeficiency virus (HIV) status is an important step in linking HIV-infected individuals to care and treatment and HIV-uninfected persons to prevention services. Early initiation of HIV treatment has been shown to reduce rates of HIV transmission, improve health outcomes and reduce cost of care [1–3]. Therefore, UNAIDS recommends the scale-up of HIV testing in countries with high HIV prevalence as part of the 90–90-90 initiative where 90% of HIV-infected people are meant to know their status [4]. Consequently, HIV self-testing has been introduced in various countries and is being assessed as a strategy to increase access and uptake of HIV testing, especially among under tested populations [5–7]. In the newly-released consolidated guidelines on HIV testing services, the World Health Organization (WHO) encourages countries to conduct pilot and demonstration projects of HIV self-testing to provide evidence on the role self-testing can play in closing the HIV testing gap [6]. Although the procedure is referred to as HIV self-testing in most implementing countries, South Africa opts to use the term HIV self-screening (HIVSS) to emphasise the need for a diagnostic and confirmatory HIV test. WHO recommends further testing by a healthcare provider for those at high, ongoing HIV risk and those with a positive HIV self-test result [6].

In 2016, South Africa had 270,000 new HIV infections and 7.1 million people living with HIV. Of those only 56% were receiving antiretroviral medication [8]. Despite the country implementing several HIV counselling and testing (HCT) models (clinic, mobile, stand-alone HCT clinics and circumcision programmes) to improve access to HIV testing, uptake remains poor, especially among men and adolescents. This is due to structural barriers, stigma and discrimination [9–11]. Following the recent pre-qualification of the OraQuick HIV self-screening kit (OraSure Technologies Inc., Pennsylvania, USA) by the WHO [6], the South African National Department of Health (DoH) is investigating the implementation of HIVSS at public health facilities and as part of programmes to overcome some of the barriers associated with the current HIV testing models [6, 12].

The objective of this study was to conduct a demonstration project on the feasibility (practicability and suitability for integration into existing community based HIV testing programs) of HIV self-screening among men, young women and sex workers in two areas in Gauteng.

## Methods

### Study setting

The study was conducted across two townships in the northern Johannesburg Metro Municipality, Gauteng. The two sites each have formal and informal settlements with an estimated population of 140,000 and 180,000 respectively in 2011. The provincial Gauteng HIV prevalence in 2012 was 12.4 and 17.6% in 2017 for adults aged 15–49 years [13–15]. HIV prevalence is disproportionately higher among females (14.4%) compared to males (9.9%), and is substantially higher in the 15–49 year age group (17.8%) compared to those aged 50 years and older (6.9%) [13].

### Study design

The HIV self-screening demonstration project was a mixed-methods study involving prospective enrolment of participants for HIV testing from May–November 2017. The study population included those at high risk of contracting HIV, such as young women aged between 18 and 35 years, sex workers, and men who do not generally access healthcare services. These townships were selected as they had an existing outreach HIV-testing programme. Eligibility criteria included being 18 years or older, residing in the two study sites, having access to a telephone and willingness to provide contact information and to share HIV status with the study staff.

### Study procedures

#### Recruitment

We used four strategies to recruit potential participants.

1. *Mobile HIV testing sites* were set up temporarily with colourful gazebos in areas with substantial numbers of passing community members such as shopping centres, college campuses and sports fields. Six sites, three per township, were erected for 5–10 days each time, depending on the demand for HIV testing services.
2. *Homebased recruitment* involved door-to-door visits in selected areas in each township based on the existing HIV-testing programm outreach schedule. Every street in the selected area was visited to recruit participants and enrol them in their homes.
3. Through *workplace testing,* two companies (a filling station and non-governmental organisation) in one township with a high number of males were approached and offered HIV counselling, screening and testing to all employees.
4. *Sex workers programme*: We collaborated with two established organisations in the two townships that provided HIV care and treatment services to sex workers to ensure representation of this high HIV risk population in the study.

Information sessions on the OraQuick HIV self-screening and HIV testing were provided to individuals and/or groups of 10 people or less. Participants not willing to use the HIV self-screening were offered an HIV rapid test according to South African HIV-testing guidelines [16].

#### HIV self-screening

A study staff member explained how to use the HIVSS test according to instructions on the package insert of the OraQuick test. The package insert was in English and was translated to other local languages. Participants were offered three different methods of self-screening:

- Supervised

A lay HIV counsellor assisted the HIV self-screener on site with all aspects of HIV self-screening: the testing procedure, interpretation of results, and the provision of pre- and post-test counselling. The counsellor then referred the participant appropriately to ensure linkage to care.

- Semi-supervised

A lay HIV counsellor assisted the self-screener on site with some aspects of HIV self-screening on request. This included the testing procedure, the interpretation of results, pre- and post-test counselling and appropriate referrals.

- Unsupervised

The participant would complete testing without any assistance from study staff. However, group or individual demonstration of HIV self-screening was provided and pre- or post-counselling was accessible, either at their homes or telephonically.

#### HIV testing

Since HIV self-screening is not recognised as a diagnostic test in South Africa, participants that opted for supervised and semi-supervised self-screening methods were also offered routine HIV rapid testing. The test strategy and algorithm for rapid testing were according to the current DoH guidelines [16]. These participants received immediate results and post-counselling. Those participants who opted for unsupervised HIV self-screening were issued with HIV self-screening kits. All participants that tested for HIV or received an HIV self-screen kit were provided with a referral letter to access further care.

#### Post-HIV screening kit distribution review

Follow-up assessments were conducted telephonically for all participants who tested HIV positive after either semi- or supervised HIV self-screening and for those that selected unsupervised HIV self-screening to evaluate linkages to care. A maximum of three telephonic follow-up calls (with the first telephonic follow-up being a week after distribution) or until the participant indicated that they used the kit or accessed a primary and confirmatory test. If three telephonic attempts at different times of the day were unsuccessful, a home visit was done. If both telephonic and home follow-up visits were unsuccessful, the participant was considered lost to follow-up. HIV-negative participants’ follow-ups were concluded once a primary HIV test was done, while HIV-positive participants’ follow-up was concluded after the primary and confirmatory test and if they were linked to HIV care and treatment.

### Data collection

Trained researchers used hard copies of structured questionnaires to administer the pre-, follow-up and post-interviews. Questions included in the pre-HIV self-screening questionnaire administered were: participant demographics; prior HIV testing; knowledge of HIV self-screening; reasons for choosing self-screening; preferred method of screening; expectations of the kit; and if they would recommend the self-screening kit to others. The follow-up questionnaire focused on HIV self-screening results; if the participants has shared their result with anyone; and if they accessed primary; and confirmatory tests. Once the participant was tested or reported using the kit, a post-test questionnaire was administered to explore their experience of using the kit; whether initial expectations were met and if they would recommend that HIV self-screening should be made available more widely. Although the questionnaire guide was in English, trained researchers used local languages (isiZulu, Sesotho, Setswana) when required. Anonymised collected data were captured into REDCap, a secure (password-protected) online application system and exported for statistical analysis [17].

### Data analysis

Data was analysed in Statistical Analysis Software SAS Enterprise Guide 7.1 (SAS Institute Inc., Cary, NC, USA). Fifteen participants with missing critical key variables (demographics, preferred method of testing) were not included in the analysis. Continuous data were analysed by medians and interquartile ranges (IQR), whereas frequencies were determined for categorical data and compared by method of HIV testing using the chi-square test of proportions. The proportion testing HIV positive using the OraQuick test was determined by the results from the supervised and semi-supervised, and self-report for those that performed unsupervised HIV self-screening.

## Results

**Fig. 1:**
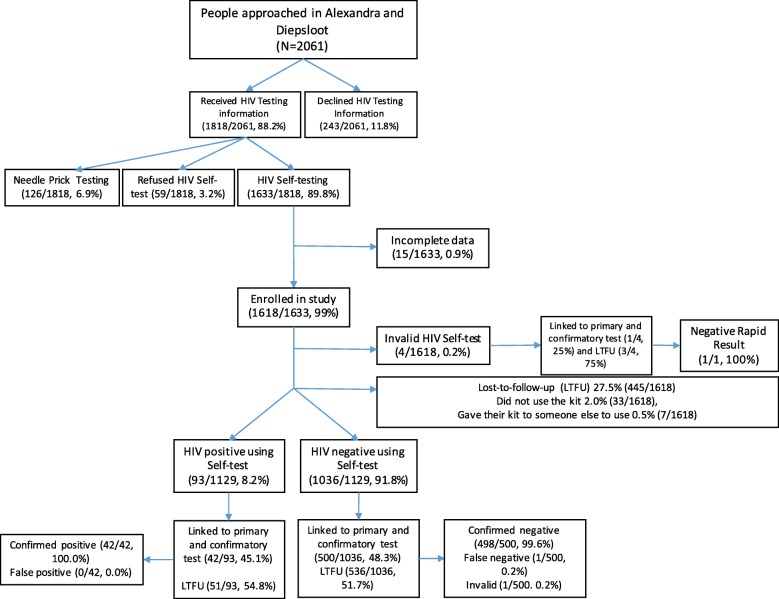
Flow chart of total HIV self-screened participants approached, refused, enrolled and HIV testing results in the feasibility of HIV self-screening study

A total of 2061 people were approached through mobile sites, their workplaces, sex worker programmes (SWP) and house-to-house recruitment for HIV testing within seven months in 2017. The majority agreed to receiving HIV-testing information (88.2%, 1818/2061) and were offered either OraQuick self-screening and standard HIV rapid test using a fingerprick. Among the people that received HIV-testing information, 89.8% (1633/1818) opted for HIV self-screening, of whom 1618 participants enrolled in the study (Fig. 1).

### Demographic characteristics of those who received a screening kit

The majority (68.3%, 1105/1618) of participants who were issued an HIV self-screening kit were older than 24 years of age (median 28 years inter quartile range 23–33).) Half the participants were male (50.0%, 808/1618) (Table 1, although we aimed to enrol more males). Two thirds (69.2%; 559/808) of the men who received an HIVSS were older than 24 years. Among the women enrolled in the study, 87.4% (707/809) were between 18 and 35 years. A total of 117 (7%) female sex workers (FSW) were enrolled. Only 17.6% (271/1542) of participants had completed high school and 36.7% (546/1486) had a monthly income of ZAR 1000 (approximately USD 75) or more.

**Table 1:** Socio-demographic characteristics of participants that received HIV self-screening kits in the feasibility of HIV self-screening study

Variable	Number	Proportion
Number enrolled for HIV self-screening	1618	
Age in years		
18–24 (%)	513	31.7%
> 24 (%)	1105	68.3%
Median age (IQR)	28.0 (23.0–33)	
Gender		
Female (%)	809	50.0%
Male (%)	808	50.0%
Place of recruitment		
Home (%)	696	43.0%
Mobile site (%)	687	42.5%
Workplace (%)	118	7.3%
Sex Workers Programme (%)	117	7.2%
Language spoken at home		
IsiZulu/IsiNdebele/IsiXhosa %)	557	35.0%
Sepedi/Sesotho/Setswana %)	520	32.7%
Xitsonga (%)	239	15.0%
Tshivenda (%)	131	8.2%
Other	144	9.1%
Highest level of education		
Primary school (%)	1271	82.4%
High school (%)	123	8.0%
Tertiary (%)	148	9.6%
Income level		
R0 - R999 (%)	940	63.3%
R1000 - R4999 (%)	378	25.4%
R5000 - R9999 (%)	134	9.0%
R10000+ (%)	34	2.3%

### Utilised screening methods

The majority of participants were recruited through homebased outreach (43.0%, 696/1618); 42.5% (687/1618) at mobile sites, 7.3%, (118/1618) the workplace, 7.2%, (117/1618) and through SWPs. Overall preferred method of HIVSS was unsupervised (68.7%, 1110/1616), followed by supervised (25.0%, 405/1616) and semi-supervised (6.3%, 101/1616). Unsupervised HIVSS was preferred across all the four recruitment strategies (Table 2), and across age groups. Few (3.8%, 42/1110) of those who initially opted for unsupervised screening subsequently requested assistance with HIVSS. Over two thirds of participants opted for unsupervised HIV self-screening, although more women than men preferred the unsupervised method of HIV self-screening (73.6%, 595/809 vs 63.8%, 515/807) and 69% of 18–24 year olds and 68% of those older than 24 years selected unsupervised HIVSS.

**Table 2:** Recruitment strategy by method of self-screening in the feasibility of HIV self-screening study

Place of recruitment	Supervised (n, %)	Semi-Supervised (n, %)	*p*-value
Homebased	126/405 (31.1)	71/101 (70.3)	< 0.0001
Mobile	234/405 (57.8)	29/101 (28.7)	< 0.0001
Workplace	39/405 (9.6)	1/101 (0.99)	0.0040
Sex work programme	6/405 (1.5)	0/101 (0)	–
	Supervised	Unsupervised	
Homebased	126/405 (31.1)	499/1111 (44.9)	< 0.0001
Mobile	234/405 (57.8)	423/1111 (38.1)	< 0.0001
Workplace	39/405 (9.6)	78/1111 (7.0)	0.0921
Sex work programme	6/405 (1.5)	111/1111 (9.99)	< 0.0001

### Self-screening HIV results

Of the 1618 individuals who chose HIV self-screening kits, 70.0% (1133/1618) used the kit, 2.0% (33/1618) reported that they did not use the kit, and 0.5% (7/1618) gave their kit to someone else to use (Fig. 1). The proportion of participants that used the HIV self-screening kit was similar (97%) among males (572/588) and females (561/578), and 45.3% (513/1133) participants were aged between 18 and 24 years. Data on usage of the kit were not available on 27.5% (445/1618) of the participants unreachable on follow-up. Of all participants who selected the unsupervised HIVSS across all the recruitment strategies and who also had at least one follow-up, 95.4% (641/672) reported using the HIV self-screening kit.

**Fig. 2:**
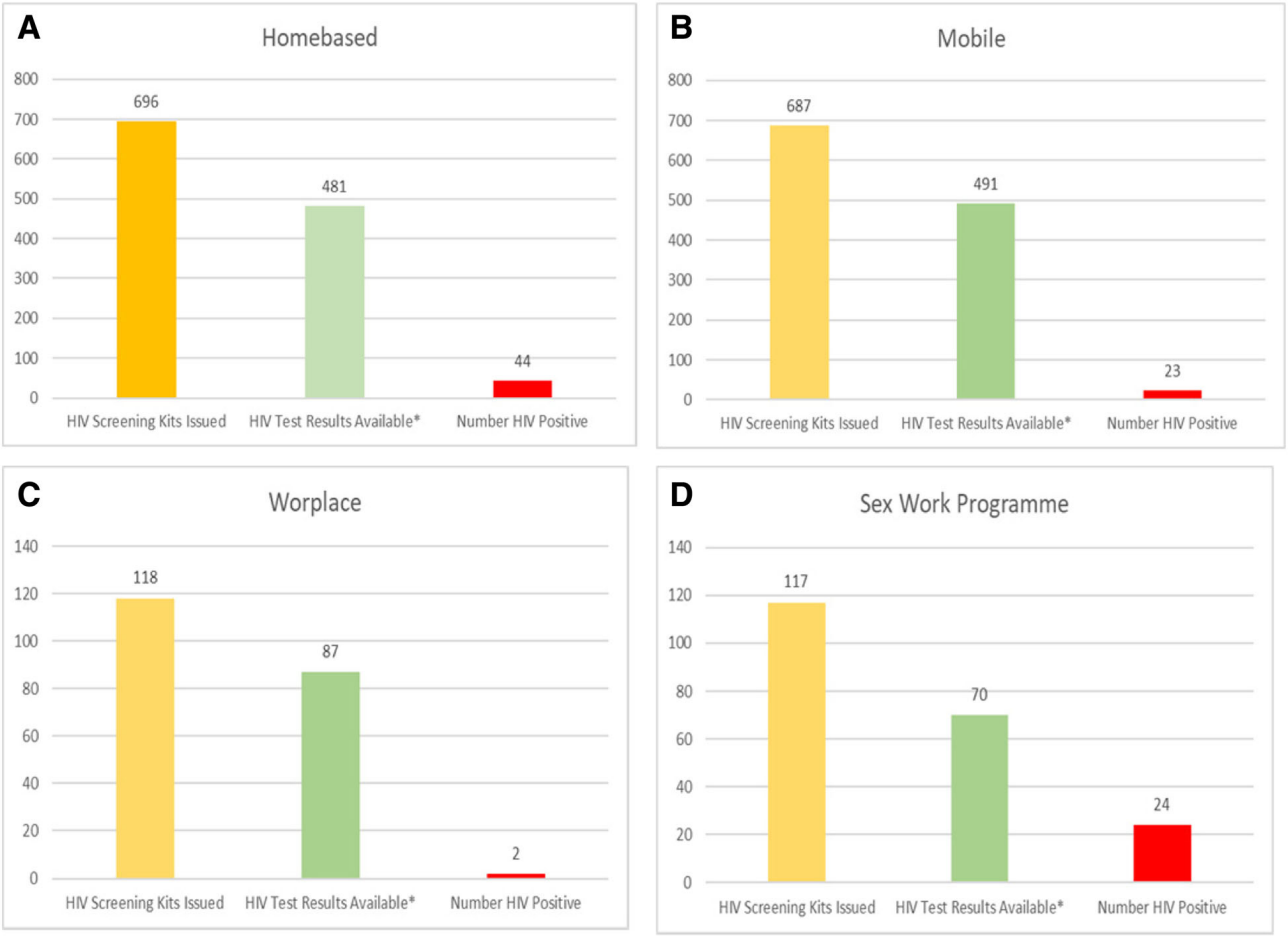
HIV screening by recruitment strategy cascade in in the feasibility of HIV self-screening study. **a**. Homebased, **b**. Mobile site, **c**. Workplace, **d**. Sex work programme

Overall, HIV prevalence based solely on the HIVSS kit was 8.2% (93/1129); and by testing strategy, unsupervised 10.0%, (64/640) compared to supervised 5.6% (22/394) and semi-supervised 7.2% (7/97). HIV prevalence based on HIVSS kits in individuals recruited through the homebased strategy was 9.1% (44/481), in the mobile site strategy 4.7% (23/491), in workplaces 2.3% (2/87) and in the FSW programmes was 34.3% (24/70) (Fig. 2). Some (19.4%; 18/93) of the participants that tested HIV positive already knew their HIV status. A small proportion of participants (0.2%, 4/1618) who opted for the unsupervised method of HIV self-screening reported obtaining an invalid (no stripe) result at follow-up interviews.

Of the participants who indicated their HIV-testing history, 90.6% (1452/1603) stated that they had previously tested for HIV. Of these 41.7% (600/1440) had tested at least one year prior to study enrolment, 53.1% (319/600) were males, and 70.3% (422/600) were > 24 years old. Participants reported government primary healthcare facilities and hospitals (55.2%, 770/1396) as the main sites where they previously tested, while others had tested at private healthcare facilities and workplace programmes.

During follow-up of the participants who reported a HIV-positive result, 50.9% (29/57) shared their results with their partners. Two (2/28; 7.1%) HIV-infected participants in the unsupervised method, reported experiencing violence after sharing their results.

### Linkage to primary and confirmatory HIV testing

During follow-up, access to post-test HIV services was assessed a week after receiving the HIVSS test kit. Participants confirmed linkage to care and treatment: counselling (8.7%, 99/1133) across all groups, initiation on antiretroviral therapy (16.0%, 12/75) and CD4-testing (5.3%, 4/75) for those screening HIV positive.

Overall, nearly half (48.0%, 543/1131) of the participants that used the HIV self-screening kit received a primary HIV rapid test using the current serial HIV testing algorithm according to the DoH guidelines [16]; overall HIV positivity prevalence in that group was 7.9% (43/543). Of the participants that received unsupervised HIVSS and reported their results, 18.1% (116/640) had a confirmatory HIV test. Most of the participants that had semi-supervised (93.8%; 91/97) and supervised (85.2% 336/394) also received a serial confirmatory HIV test.

## Discussion

In this study, HIVSS using the OraQuick assay self-screening through homebased, mobile site, workplace and sex work programmes was feasible and the preferred method of HIVSS was unsupervised compared to semi-supervised and supervised methods of HIVSS. HIV self-screening appeared to facilitate access to populations at high risk of HIV infection, and to people who had not tested for HIV within the past year.

Other studies of HIV self-screening have also included high-risk population groups such as men having sex with men (MSM), pregnant women, healthcare workers and injecting drug users [18]. However, this is one of few studies to include sex workers and men over the age of 24 years. In this study, half of the participants were men, who many studies have reported to have a lower uptake of HIV testing [19]. An HIV self-testing study conducted in Malawi also reported similar proportions of both genders being tested, despite fewer men reporting previous HIV testing [18]. Although we included apparently high risk populations, overall prevalence was 8.2% in these adults(18–70 years), while the Gauteng provincial HIV prevalence in 2017 was 17.6% in adults aged 15–49 years [15].

There was a high proportion (88%) of people who were interested in receiving HIV testing information and virtually all wanted to try HIVSS. Our findings are similar to those of previous studies [5, 18, 20, 21] that reported a willingness to use HIV self-testing kits of up to 87% [5].

Linkage to primary and confirmatory testing was low despite participants being issued with referral letters on enrolment. Although data were not collected on reasons for failure to access additional HIV services in this study, reasons mentioned in other studies include no integration of HIV and primary healthcare services, travel costs, family responsibility and substance abuse [22].

### Strengths and Limitations

This study was a field demonstration project designed to asses feasibility and attempting to replicate conditions of existing primary healthcare programmes and other initiatives to increase access to HIV testing. The study also assessed different recruitment strategies (homebased, mobile, workplace and SWPs), which were successful as the study accessed high-risk groups (sex workers, young women, men over the age of 24 years). The test kits used in the study were pre-qualified by the WHO.

Participants in our study refusing HIV self-screening were not followed up to assess their linkages to other healthcare services. People approached for participation in the study received information on the HIV self-screening method from the study team, which could have biased their perceptions and uptake in favour of HIVSS. Furthermore, the HIVSS uptake could have been lowered by the requirement to have rapid HIV finger-prick test after HIV self-screening. The work place recruitment strategy proved to be challenging as it would have required work schedules to be suspended to accommodate the information session provided by the study team.

The overall follow-up in the study was low as many participants could not be reached over the phone or through home visits. This high rate of lost-to-follow-up might have resulted in underreporting of HIV diagnosis, as well as participants not being interested in going to healthcare facilities to get tested for HIV again. The study did not assess linkages to HIV prevention services for participants that tested HIV negative and at high risk of contracting HIV.

## Conclusion

Linkages to confirmatory testing and further care were relatively low amongst those that opted for unsupervised HIVSS. Potential approaches to scale up and accelerate HIV self-screening integration should consider distribution methods, increasing awareness, cost and cost effectiveness implications, and strengthening linkages to confirmatory testing and further care possibly using mobile teams to travel to those that test HIV infected. HIV self-screening was feasible and easily implemented through homebased, mobile site, workplace and SWPs and unsupervised was the most preferred and utilised HIV self-screening method. The study was successful in reaching young women, men over the age of 24 years and sex workers across the two regions.

## Data Availability

The datasets generated and/or analysed during the current study are not publicly available due to specifications by the ethical review board. However, they are available from the corresponding author on reasonable request.

## Abbreviations

DoH: South African National Department of Health
HIV: Human immunodeficiency virus
HIVST: HIV self-testing
SWP: Sex workers programmes
WHO: World Health Organization
